# Cardiolipin Stabilizes and Increases Catalytic Efficiency of Carnitine Palmitoyltransferase II and Its Variants S113L, P50H, and Y479F

**DOI:** 10.3390/ijms22094831

**Published:** 2021-05-02

**Authors:** Beate Meinhardt, Leila Motlagh Scholle, Franziska Seifert, Martina Anwand, Markus Pietzsch, Stephan Zierz

**Affiliations:** 1 Department of Neurology, Martin-Luther-University Halle-Wittenberg, Ernst-Grube-Str. 40, 06120 Halle (Saale), Germany; leila.scholle@medizin.uni-halle.de (L.M.S.); stephan.zierz@medizin.uni-halle.de (S.Z.); 2 Department of Pharmaceutical Technology and Biopharmacy, Institute of Pharmacy, Martin-Luther-University Halle-Wittenberg, Weinbergweg 22, 06120 Halle (Saale), Germany; franziska.seifert@pharmazie.uni-halle.de (F.S.); martina.anwand@pharmazie.uni-halle.de (M.A.); markus.pietzsch@pharmazie.uni-halle.de (M.P.)

**Keywords:** carnitine palmitoyltransferase II, cardiolipin, thermostability, S113L, P50H, Y479F, protein stability, muscle disease, CPT II deficiency

## Abstract

Muscle carnitine palmitoyltransferase II (CPT II) deficiency is associated with various mutations in *CPT2* gene. In the present study, the impact of the two CPT II variants P50H and Y479F were characterized in terms of stability and activity in vitro in comparison to wildtype (WT) and the well investigated variant S113L. While the initial enzyme activity of all variants showed wild-type-like behavior, the activity half-lives of the variants at different temperatures were severely reduced. This finding was validated by the investigation of thermostability of the enzymes using nano differential scanning fluorimetry (nanoDSF). Further, it was studied whether the protein stabilizing diphosphatidylglycerol cardiolipin (CL) has an effect on the variants. CL indeed had a positive effect on the stability. This effect was strongest for WT and least pronounced for variant P50H. Additionally, CL improved the catalytic efficiency for CPT II WT and the investigated variants by twofold when carnitine was the varied substrate due to a decrease in K_M_. However, there was no influence detected for the variation of substrate palmitoyl-CoA. The functional consequences of the stabilization by CL in vivo remain open.

## 1. Introduction

**Figure 1:**
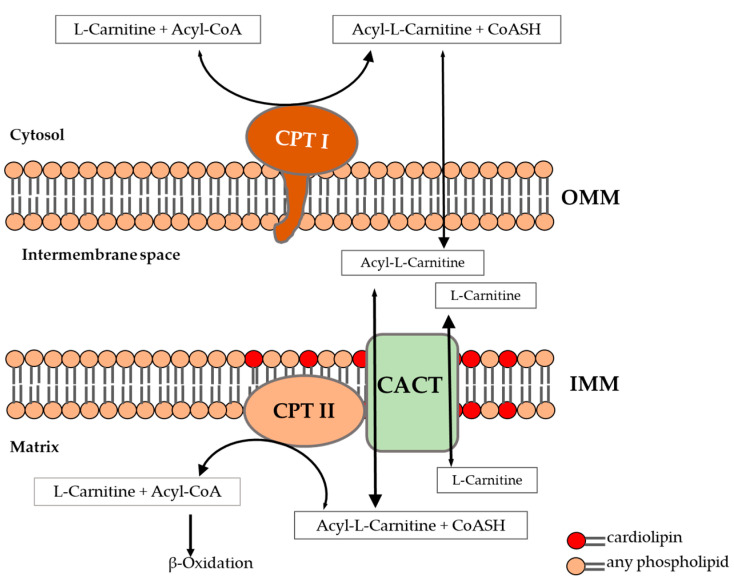
Transport of fatty acids through mitochondrial membrane via carnitine shuttle modified according to McGarry [3]. CPT, carnitine palmitoyltransferase, CACT, carnitine:acylcarnitine translocase, CoA, Coenzyme A, IMM, inner mitochondrial membrane OMM, outer mitochondrial membrane.

The β-oxidation of long-chain fatty acids (LCFAs) is the major source of energy for skeletal muscle. The entry of LCFAs into the mitochondria requires a carnitine-dependent transport system [1] (Figure 1). The so-called carnitine shuttle consists of carnitine palmitoyltransferase I (CPT I), carnitine:acylcarnitine translocase (CACT) and carnitine palmitoyltransferase II (CPT II). CPT I is an integral outer mitochondrial membrane protein and converts acyl-CoAs to acylcarnitines, which are released into the intermembrane space. Acylcarnitines are translocated into the mitochondrial matrix by CACT in exchange with unesterified carnitine. CPT II, located on the inner mitochondrial membrane, catalyzes the reconversion of acylcarnitines to acyl-CoAs, thereby providing substrates for β-oxidation [1,2].

CPT II is a homotetrameric protein [4,5], associated with CACT [6] and loosely associated with the inner mitochondrial membrane (IMM) [2,7].

The major phospholipids of the IMM of mammalians are phosphatidylcholine (40%), phosphatidylethanolamine (30%) and cardiolipin (CL 15–20%) [8]. CL is involved in various mitochondrial processes such as bioenergetics, cristae morphology and supercomplex formation [9,10,11] and has an amphiphilic character. Unlike other phospholipids, the diphosphatidylglycerol CL has a unique structure consisting of highly specific acyl chains. The structure and composition of acyl chains seems to play a crucial role in the importance of CL [12]. It has been reported that CL is essential for the activity and stability of CACT, a part of the carnitine shuttle [13,14,15].

A possible association of CPT II with CL was predicted on the basis of structural and computer-based analyses [16] and an increased enzyme activity, and a protein-phospholipid interaction was reported for a recombinant rat CPT II in presence of CL [17]. CPT II retains its enzymatic activity after rupturing the mitochondrial membrane [2].

Thus far, more than 60 mainly pathogenetic mutations in the *CPT2* gene have been reported [18]. The CPT II deficiency is the most frequent deficit of mitochondrial fatty acid β-oxidation in skeletal muscle and is inherited autosomal recessively [19]. There are three clinical phenotypes: (i) infantile; (ii) neonatal; and (iii) muscular or myopathic form. The muscular presentation is the most common and less severe phenotype and characterized by recurrent episodes of myalgia, muscle weakness and rhabdomyolysis, which can lead to renal insufficiency in more severe cases [20]. The muscular form most frequently occurs in teenagers and/or young adults [19,20,21]. In most of the cases described, the main triggering factor for the attacks was prolonged exercise [22]. Additionally, exposures to cold, fever and infections were described as triggering parameters as well [23]. All these events have the increase in body temperature in common. [24,25,26,27,28]. It is therefore assumed that the symptoms of muscle phenotype in patients with CPT II deficiency correlate with the change in body temperature [29].

Various mutations in *CPT2* gene with different origins of enzyme domains lead to the muscle phenotype of CPT II deficiency. The most frequent enzyme variant is S113L [21,23,30]. This mutation is located in the amino-terminal domain near a substrate binding domain of the enzyme [30,31]. Malonyl-CoA is an important regulator of LCFA transport into the mitochondria by inhibiting CPT I. CPT II WT is not inhibited by this compound [32]. The amino acid exchange S113L oft CPT II leads to an abnormal enzyme inhibition by malonyl-CoA *in vitro.* In addition, marked thermosensitivity of this variant was detected [29]. This was interpreted as a possible cause for the provocation of symptoms. The second frequent variant causing a muscle phenotype is P50H [22,33,34], located near the membrane associating domain [30] at the amino-terminal domain. A possible interference with the association between CPT II and the mitochondrial membrane caused by this amino acid exchange was suggested [34]. An altered stability of this CPT II variant was also suspected [35]. The variant Y479F was identified as a private mutation [33] also causing a muscle phenotype. In contrast to the localization of S113L and P50H, this amino acid exchange is located at the carboxy-terminal domain [30].

The biochemical consequences of the CPT II variants P50H and Y479F including the effect of CL have not been characterized so far. In the present study, the variants P50H and Y479F were compared to WT and variant S113L *in vitro*. Additionally, the influence of CL on CPT II and its variants was studied because previously stabilizing effect of CL on CACT has been reported [13]. The kinetic behavior of the enzymes was investigated using the substrates palmitoyl-CoA (Pal-CoA) and L-carnitine and the well-known competitive inhibitor R-amino carnitine. The CPT II variants clearly differed in their thermostability with variant P50H as the most thermosensitive among the enzyme variants. Both the temperature dependent enzyme activity and the protein stability could be modulated by the amphiphilic substance CL.

## 2. Results

### 2.1. Thermosensitivity of CPT II Variants

To evaluate the enzyme behavior under different temperature conditions that are relevant under physiological conditions, we chose 30 (cold), 37 (normal), 40 (moderate fever) and 42 °C (high fever) for the experiments. The enzyme variants were expressed recombinantly in *E. coli*, carried a N-terminal histidine-tag and were purified by standard chromatography in the presence of a detergent (for experimental details, see Section 4.1 and Section 4.2). The impact of temperature was investigated by incubating purified CPT II variants at appropriate temperatures and determining their enzymatic activity after defined incubation periods. The enzyme assay was based on an adaptation of Rufer et al. [30] for the transfer reaction of the palmitic acid from palmitoyl-CoA to L-carnitine (see Section 4.3).

**Figure 2:**
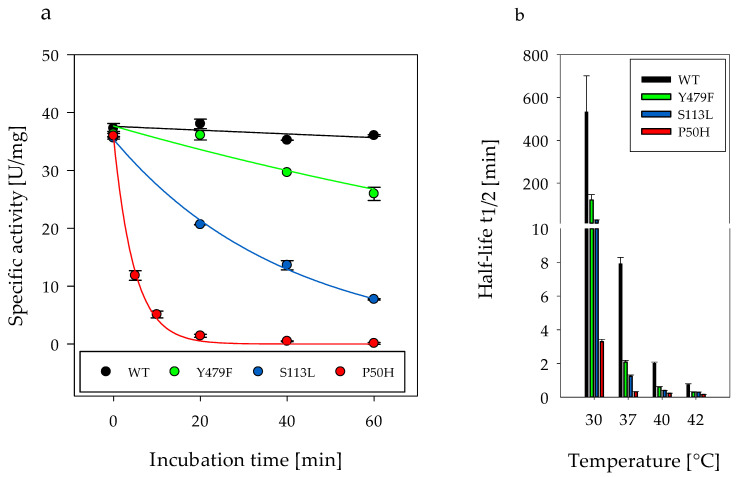
(**a**) Specific activity of recombinant CPT II wild type and variants (enzyme concentration 1 mg/mL during incubation time) at 30 °C measured spectroscopically using enzyme assay. The reaction was started by adding 15 nM enzyme. (**b**) Half-lives of wild type and variants (1 mg/mL) at different temperatures. Error bars are standard deviation (SD) derived from three independent experiments. The statistical differences of the half-lives between WT and the variants and between the different temperatures are given as *p*-values in Tables S1 and S2.

The initial enzymatic activities of the variants were similar to the wild type (WT) (Figure 2a, incubation time 0 min). WT showed stable activity at an incubation concentration of 1 mg/mL at 30 °C over 60 min. However, the activity of Y479F, S113L and P50H decreased during incubation time already at 30 °C (Figure 2a), with complete loss of activity for variant P50H within 25 min. Increasing temperatures up to 42 °C correlated with shorter half-life of enzyme activity for all variants compared to WT (Figure 2b and Tables S1 and S2). At 37 °C, the half-life of Y479F activity was only a quarter that of WT and the half-lives of the other variants were even lower. The variant P50H had the shortest half-life under all conditions tested.

After and during heat treatment, aggregation along with inactivation was observed. The aggregation of CPT II WT and variants was also visible in the aggregation optic measurement during the nanoDSF experiment (Figure S1).

### 2.2. Effect of Cardiolipin on Thermosensitivity of the Enzyme Activity

Then, the effect of CL, as a potential stabilizing agent, was investigated in the activity assay. Therefore, 0.1 mg/mL recombinant enzyme was incubated with various concentrations of CL (0.125, 0.25 or 0.5 mM) or buffer at 30 and 42 °C, respectively.

**Figure 3:**
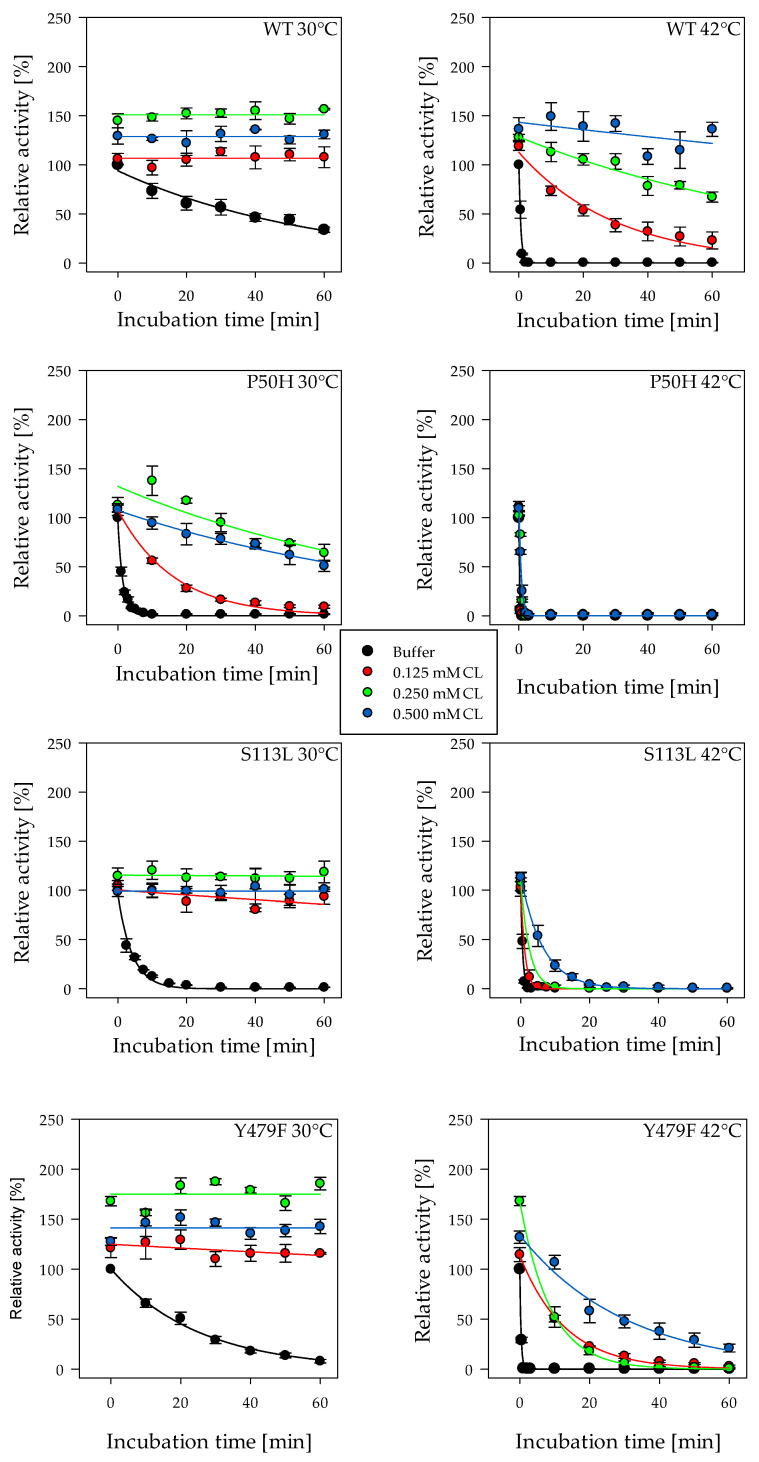
The effect of varying cardiolipin (CL) concentration on the enzymatic activity of recombinant CPT II (enzyme concentration 0.1 mg/mL during incubation time) after incubation at 30 and 42 °C. The enzyme assay was started by adding 15 nM enzyme to the reaction mixture. Black symbols represent the relative activity values after incubation with buffer. Red, green and blue symbols represent the relative activity values after incubation with 0.125, 0.25 and 0.5 mM CL, respectively. Enzyme activity was measured at 25 °C as described before [32]. The initial activity value without incubation was set to 100%, respectively. WT, wild type; variants Y479F, S113L and P50H. Error bars are SD derived from three independent experiments. The statistical differences of the maximal enzymatic activities between measurements in buffer or buffer with cardiolipin are indicated as *p*-values in Tables S3 and S4.

The addition of CL increased the enzyme activity for all tested enzyme variants before subsequent incubation periods. (Figure 3 and Tables S3 and S4). Enzymatic activity of WT increased significantly in the presence of 0.25 and 0.5 mM CL up to 150%. For variant Y479F (175%), this effect was even more pronounced. The increase in enzyme activity was less noticeable for variants S113L (115%) and P50H (113%). Furthermore, the presence of CL resulted in maintenance of CPT II enzyme activity at both temperatures compared to enzyme/buffer samples even at low CL concentrations during the tested period of time. At 30 °C, 0.25 mM CL had the greatest stabilizing effect, but this impact shifted to 0.5 mM CL at 42 °C. Interestingly, the positive influence on the WT was stronger than on the variants at higher temperatures (Figure 3, 42 °C). At 42 °C, CL showed almost no effect on variant P50H. Confirming the initial experiment again, variant P50H showed the highest thermosensitivity, followed by S113L, Y479F and WT (Figure 3).

### 2.3. Effect of Cardiolipin on the Protein Stability

Based on our findings that all amino acid exchanges showed moderate to severe thermosensitivity and the fact that CL displayed stabilization behavior under reaction conditions, it was interesting to further characterize the thermostability of the enzymes. Thus, the protein stability was investigated applying nano differential scanning fluorimetry (nanoDSF) using 1 mg/mL protein in absence or presence of 0.25 mM CL (for details, see Section 4.5). NanoDSF is a standard method for the investigation of the folding/unfolding of proteins and the interaction of proteins with effectors/ligands/substrates [36,37]

**Figure 4:**
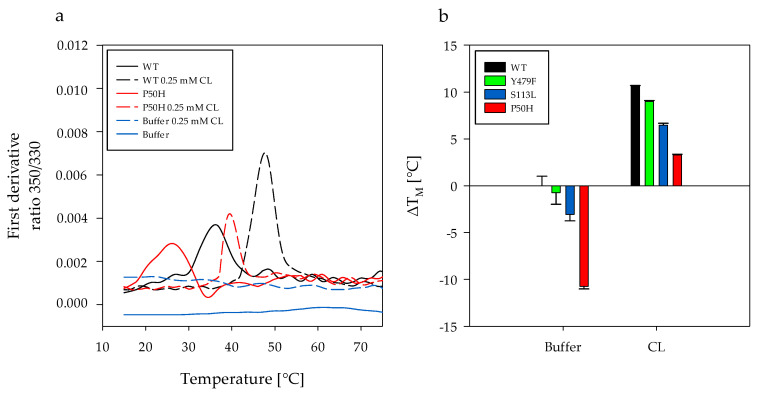
The effect of amino acid exchange and the addition of 0.25 mM CL on the stability of CPT II (1 mg/mL) assessed by nanoDSF. (**a**) The first derivatives of the transition curves resulting from the ratio 350/330 nm were plotted against the temperature. The T_M_ values are derived from the maxima of the obtained curves. The black and red solid lines show the curves of WT and variant P50H measured in assay buffer. The black and red dashed lines show the results of WT and variant P50H measured in assay buffer with 0.25 mM CL. (**b**) The T_M_ of WT (37 °C) in buffer was used as reference value (ΔT_M_ = 0 °C) to determine the ΔT_M_ for the variants and the ΔT_M_ for WT and variants after the addition of 0.25 mM CL. Error bars are SD derived from three independent experiments. The significant differences of ∆T_M_ of WT and the variants between the different buffer composition as well as the statistical differences of T_M_ between WT and the variants are indicated as *p*-values in Tables S5–S7.

The transition curves of WT and variant P50H in presence (dash lines) and absence (solid lines) of CL are shown exemplarily in Figure 4a as first derivatives. The maxima of the black and red graphs display the transition temperature (T_M_) of WT and variant P50H, respectively. The T_M_-value of WT in absence of CL (Figure 4a, solid line, 37 °C) was used as reference for comparing WT and variants and the effect of CL on protein stability (Figure 4b). The results of the temperature transition from 15 to 95 °C revealed lower T_M_-values for all variants compared to WT (Figure 4b). While the difference was moderate for variants S113L and Y479F, ΔT_M_ was as large as 10 °C for variant P50H. The addition of CL was able to increase the T_M_ value for all enzymes (Figure 4b and Tables S5–S7). The strongest effect was detectable for variant P50H (ΔT_M_ = +13 °C). For WT and the other variants, the ΔT_M_-value increased by +10 °C due to the addition of CL. Despite the increased stabilities of the CPT II variants, the stability of WT could not be achieved.

As shown in Figure 4a, the addition of CL also leads to a doubling of the first derivative values at the maxima of WT and variants. This could indicate a change in protein conformation in the microenvironment of the intrinsic proteinogenic fluorophores tryptophanes and tyrosines upon interaction with CL. This consequently would strengthen the hypothesis of stabilization. Furthermore, the maximal values of the first derivative of the variants S113L and Y479F were similar (Figure S2). This value is slightly lower for WT and significantly reduced for variant P50H, indicating that the amino acid exchange P50H has an impact on the CPT II structure.

These findings support the data from the enzyme activity measurements in terms of thermosensitivity of the variants as well as the stabilizing effect of CL.

### 2.4. Effect of CL on the Catalytic Efficiency of CPT II

#### 2.4.1. Conversion of Palmitoyl-CoA

To gain further insights into the details of the positive effect of CL the kinetic parameters, *k_cat_* and K_M_ were investigated in the absence and presence of CL first varying the substrate palmitoyl-CoA (Pal-CoA). The results on the catalytic efficiency (*k_cat_*/K_M_) are displayed in Table 1. CPT II WT and the variants P50H and Y479F had comparable catalytic efficiencies with only marginal differences in *k*_cat_ and K_M_ for Pal-CoA. Variant S113L showed a 1.6-fold lower catalytic efficiency for Pal-CoA compared to WT, which results from an increase in K_M_ (Table 1). This suggests that binding of Pal-CoA might be impaired in S113L, confirming previous structural data which categorize variant S113L involved in substrate binding [32]. The addition of CL had no significant impact on the catalytic efficiency of Pal-CoA (Table 1).

#### 2.4.2. Conversion of L-carnitine

Comparable to Pal-CoA, next the kinetics were analyzed varying the concentration of the second substrate L-carnitine. The obtained data show no significant differences between WT and variants regarding the catalytic efficiency for L-carnitine. However, the use of CL increased the catalytic efficiency of all studied CPT II enzymes for L-carnitine significantly by two- to threefold (Table 2). The reason for this increase clearly was the improvement in substrate binding. The K_M_ value for L-carnitine was lowered in the presence of CL while *k*_cat_ remained unaffected.

### 2.5. Effect of CL on the Inhibition by R-amino Carnitine

CL showed effects on protein stability as well as the K_M_ value of L-carnitine. In the next experimental set up, its influence on the enzyme inhibition was analyzed. The competitive inhibitor R-amino carnitine was chosen for this purpose. The results reveal that the K_I_ values of the CPT II variants differed from WT. The data show that R-amino carnitine is a stronger inhibitor of variants P50H and S113L since the inhibition constant were threefold lower. In the case of variant Y479F, the inhibition was 1.5-fold weaker than for the WT enzyme. This suggests different impact of the individual amino acid exchanges in CPT II on the interaction with the inhibitor.

The addition of CL led to a reduction in the K_I_ values of all CPT II enzymes significantly, whereby the K_I_ values of variant P50H and S113L were half as high as WT and variant Y479F (Table 3).

### 2.6. Lipid Binding Assay

The possible binding of human CPT II to components of the mitochondrial membrane was investigated using membrane lipid strips. This experiment allows the differentiation of the interaction of the different CPT II variants to a certain type of phospholipids. Hereby, deeper insights into the influence of the variants on this interaction are possible.

**Figure 5:**
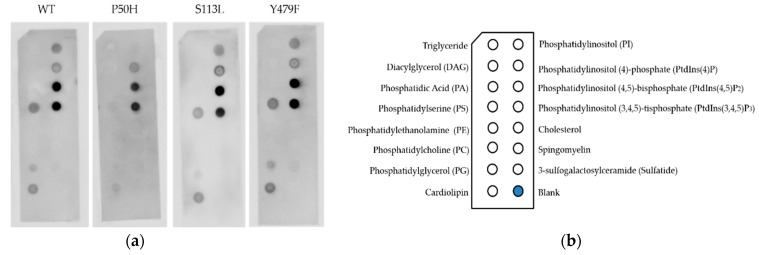
Interaction of recombinant CPT II WT and variants S113L, P50H and Y479F with phospholipids of the mitochondrial membrane. (**a**) Binding of CPT II WT and variants P50H, S113L and Y479F (final enzyme amount 0.4 mg) to lipids on membrane lipid strips. (**b**) Schematic diagram of immobilized phospholipid species on membrane lipid strips. The experiment was performed at least three times independently.

The highest signal for interaction was detected for phosphatidylinositol (4,5)-bisphosphate (PtdIns(4,5)P2) and phosphatidylinositol (3,4,5)-trisphosphate (PtdIns(3,4,5)P3) for all CPT II enzymes tested (Figure 4). The signal of interaction with CL was lowest for the variant P50H. No signal could be detected for the interaction of variant P50H and phosphatidylinositol (PI), whereas for WT and the two other variants an interaction with PI was detected. The interaction with phosphatidylserine (PS) was also reduced for variant P50H compared to WT and the other variants. Both variants S113L and P50H showed a lower detection signal for phosphatidylglycerol (PG) compared to WT and variant Y479F (Figure 5).

## 3. Discussion

The present study focused on different *CPT2* mutations that lead to muscular form of CPT II deficiency, the most common defect of lipid metabolism in skeletal muscle [2]. The resulting amino acid exchanges S113L, P50H and Y479F of the human CPT II are located in different domains of the enzyme but express similar phenotypes [22,33,34].

The initial enzyme activities of the investigated variants were similar to WT, which has already been demonstrated for the recombinant variant S113L [29]. Thus, the previous hypothesis of reduced enzyme activity [38] could also not be supported for variants P50H and Y479F. In vitro the recombinant variants showed a more pronounced thermosensitivity compared to WT, with variant P50H reacting most sensitive to elevated temperatures. Consequently, the amino acid exchanges seem to cause a destabilization of the CPT II structure without major impact on enzymatic activity itself. For variant Y479F, the reduction in enzyme activity due to prolonged incubation at different temperatures was less pronounced than for variant S113L and P50H. Thus, variant Y479F presumably has a lower impact on the stability of the enzyme than the other two variants. Based on the low impact on the CPT II stability, it could be speculated that many patients expressing the CPT II variant Y479F have not been diagnosed today. Other exclusively thermosensitive polymorphic CPT II variants with normal enzymatic activity reported only in Japanese cohorts, e.g., V368I and V605L, have been identified in patients with a more severe course of influenza-associated encephalopathy (IAE) [39,40]. Prior to the manifestation of IAE, these patients had no symptoms of CPT II deficiency [40].

The lower stability of the variants P50H, S113L and Y479F compared to WT was also confirmed by the nanoDSF measurements analyzing the melting behavior of the enzymes (Figure 4). According to these results of in vitro measurements, the reason for clinical symptoms does not seem to be a decreased or lacking enzyme activity, but a lower thermal resistance of CPT II caused by the studied amino acid exchanges, especially in the case of P50H. CPT II deficiency is characterized by recurring attacks of myalgia and muscle weakness. The symptoms are triggered by physical stress, fever, infection or exposure to cold [23]. The time dependent thermal sensitivity in case of S113L and P50H in vitro could be an explanation for the attack-like symptoms provoked by increase of body temperature (e.g., fever and prolonged exercise) [22,23] found in respective patients. Under normal conditions, the unaltered enzyme activity of the variants allows normal mitochondrial metabolism. Due to stress-related changes such as the above-mentioned situations, the variants might lose activity at least partly because of their increased thermosensitivity in comparison to the WT. Patients carrying the respective mutations are cannot cope with the altered metabolism in the muscles leading to the common episodic symptoms. However, it has to be considered that compared to experiments performed in vitro also other factors and the combination thereof, e.g., interaction with substrates/products/effectors (as shown for the inhibitor R-amino-carnitine and lipids) or other membrane-bound interaction partners such as CACT, might be involved *in vivo*, in complex pathophysiological processes.

For variant S113L, the thermosensitivity compared to WT has already been shown for elevated temperatures >40 °C, but not for 30 °C [29]. The decrease of S113L activity at 30 °C (Figure 2a) and the reduced half-lives of WT at 37–42 °C can be explained by the buffer composition used. Previously, the detergent n-octyl-β-d-glucopyranoside (β-OG) of the storage buffer was not removed indicating a stabilizing effect as well. This effect could be proven by nanoDSF experiments (Figure S3). The core statement remains the same, namely that variant S113L is more thermosensitive than WT.

In contrast to other acyltransferases which are integral or soluble enzymes, CPT II is membrane associated. This association is caused by a sequence of 30 amino acid forming a pair of antiparallel helices with membrane interacting features [30]. It was assumed that the surface properties of CPT II are eminently suitable for an interaction with CL [30]. It is well known that CL interacts with various proteins of the inner mitochondrial membrane and is necessary for their stability and enzyme activity [13,14,41]. The binding of the related mammalian CPT II from *Rattus rattus* to CL and other phospholipids as well as the increase in activity resulting in interaction of recombinant rat CPT II and CL was already shown [17]. In a previous study an activity-increasing effect of CL on recombinant human CPT II WT was verified, but no benefit for variant S113L was observed [42]. Our results of the enzymatic activity measurements as well as the investigation of protein stability clearly demonstrate the stabilizing impact of CL for WT and variants (Figure 3 (30 °C) and Figure 4). The maintenance effect for CPT II activity by the addition of CL decreased with incubation at higher temperatures simulating fever. Especially the activity of variants P50H and S113L decreased rapidly despite addition of CL during incubation at 42 °C (Figure 3). This finding also supports the fact that symptoms of CPT II deficiency are triggered by events that result in an increase in body temperature [24,25,27] and do not occur permanently in patients. Furthermore, a considerable increases of the enzymatic activity for WT (1.5-fold) and variant Y479F (1.8-fold), but only a marginal effect for variants P50H and S113L, were observed. Based on the current knowledge, it could be speculated that the lower enzymatic activity increase of the variants has pathological consequences, especially in situations with increased energy requirements.

The further investigations of the effect of CL on the kinetic parameter of CPT II WT and its variants revealed some interesting results. In the two-substrate reaction, no effect was detectable for the first substrate Pal-CoA. In case of the second substrate L-carnitine, the K_M_ value was decreased by a factor of 2–3, resulting also in a two- to three-fold increase in catalytic efficiency independent of the CPT II variant. These data suggest that CL has a positive effect on binding of L-carnitine, thereby improving the catalysis. In addition, this result was confirmed by the impact of CL on the inhibitory constant of R-amino carnitine. For both WT and variants, the K_i_ value decreased significantly showing improved binding of the inhibitor. Therefore, it can be assumed that CL affects the structural organization of the carnitine binding pocket. In future studies structural analysis, e.g., X-ray crystal structures, in presence and absence of CL could give deeper insights into this behavior.

In the lipid binding assay, the interaction of CPT II WT and the variants was shown for different phospholipids that are mainly found in the IMM (Figure 5). The binding to CL was also clearly proven even though the signal was weaker than for the other phospholipids. It might be an interesting aspect to evaluate the effect of other phospholipids for instance phosphatidylinositol derivates on activity of CPT II enzymes in further studies. Nevertheless, this result supports the stabilizing effect of CL shown in the previous experiments. WT and variants S113L and Y479F showed a similar binding pattern while variant P50H revealed an altered signal pattern for the tested phospholipids. The mutation P50H is in the vicinity of the amino acid insertion that is supposed to cause the membrane association [35]. The exchange P50H seems to cause interference with the association to CL and other phospholipids of the mitochondrial membrane, whereas variants S113L and Y479F appear not to affect membrane association.

Considering the positive effect of CL on the variants in the present study together with the known stabilizing effect of the natural substrates acyl-carnitines with middle chain fatty acids [29], it might be interesting to evaluate the effect of the combination of CL with these middle chain fatty acids. In addition, it would be of value to further investigate the influence of the amino acid exchange P50H on the interaction with CACT.

## 4. Materials and Methods

### 4.1. Generation of CPT II Variants S113L, P50H, Y479F

The CPT II WT (His_6_-N-*h*CPT2) and variants P50H (His_6_-N-*h*CPT2/P50H), S113L (His_6_-N-*h*CPT2/S113L) and Y479F (His_6_-N-*h*CPT2/Y479F) were derived using the Q5 site-directed mutagenesis kit (New England Biolabs Inc, Ipswich, MA, USA). Plasmid pET28a (+) [32] carrying humane *CPT2* WT cDNA served as template for site-directed mutagenesis. The implementation was carried out according to manufacturer’s specifications. The primer pairs listed in Table 4 were used. The nucleotide sequences of the generated expression plasmids carrying the cDNA of the investigated CPT II variants were confirmed by DNA sequencing.

### 4.2. Recombinant Expression and Purification of CPT II WT and Variants

The recombinant protein production of CPT II was performed in *Escherichia coli* (*E. coli*) BL21Gold DE cells. The transformed *E. coli* cells were cultivated at 37 °C in complex medium (50 g/L yeast extract, 0.5 g/L ammonium chloride, 20 g/L glucose, 11 g/L dipotassium hydrogen phosphate and 0.68 g/L magnesium sulfate) containing kanamycin (50 µg/mL) in a total volume of 15 L. After 4 h, the temperature was reduced to 20 °C by cooling down the fermentation vessel and CPT II expression was induced by the addition of IPTG (1 mM). To maintain sufficient supply with glucose the batch fermentation was supplemented with pulse feeds when the glucose concentration fell below 5 g/L. The first pulse (150 mL of a stock solution of 500 g/L) was given at 4.5 h of cultivation. The second pulse (100 mL of 500 mL stock solution of 500 g/L) was fed after 6.5 h of cultivation. The *E. coli* cells were harvested 4.5 h after induction by centrifugation at 4000× *g*, 4 °C for 15 min. A typical harvest of 15 L resulted in 1.5 kg biomass that was stored at −80 °C. The purification of CPT II enzymes was performed as described previously [32] using standard chromatographic separation techniques. The storage buffer contained 0.01 g/L n-octyl-β-d-glucopyranoside (β-OG), a micelle forming detergent, to stabilize CPT II enzymes. The CPT II containing aliquots were frozen in liquid nitrogen and stored at −80 °C. The purity of enzymes was monitored by SDS-PAGE. In addition, the amino acid sequences of the purified CPT II enzymes (WT and variants) were validated by electrospray ionization mass spectrometry.

### 4.3. Enzyme Activity Assay

Before activity measurements, the storage buffer of the enzymes was replaced with assay buffer (50 mM KH_2_PO_4_, 120 mM KCl, 1 mM EDTA-Na_4_) using vivaspin turbo 4 tubes (Satorius Stedim biotech GmbH, Göttingen, Germany) at 4 °C. The pH of assay buffer was adjusted with KOH to 7.4. The storage buffer was diluted at least 1:500 in assay buffer. In addition, before each experimental set up, the protein samples were centrifuged at 16,000× *g* for 5 min at 4 °C to remove any micro-aggregates. After that, the protein concentration was determined using bicinchoninic acid (BCA) protein assay kit (Thermo Fisher Scientific GmbH, Berlin, Germany). The enzyme activity of recombinant CPT II was performed following Rufer et al. [30] with modifications described elsewhere [32]. The reaction mixture had a total volume of 1 mL and comprised of assay buffer, 1 mM 5-5′-dinitro-bis-(2-nitrobenzoic acid) (DTNB), 30 μM Pal-CoA and 12 mM L-carnitine, unless specified otherwise. After pre-conditioning the mixture to 25 °C for 2 min, the reaction was started by adding 15 nM of recombinant enzyme. The reverse reaction of CPT II was detected with the enzyme activity assay. CPT II catalyzed the transfer of fatty acid residue from Pal-CoA to L-carnitine with release of CoA and Pal-carnitine. The released CoA was then detected by the reaction of the free thiol group with DTNB, forming 5-mercapto-(2-nitrobenzoic acid). This compound has a molar extinction coefficient of 13,600 M^−1^ cm^−1^ at a wavelength of 410 nm [43], and its formation was followed spectroscopically. The reaction was observed for 120 s at 25 °C. The slope of the progress curve was used to determine the reaction velocity.

### 4.4. Influence of Temperature and CL on the Enzyme Activity

The purified enzymes were incubated at different temperatures (30, 37, 40 and 42 °C) using a protein concentration of 1 mg/mL during the incubation periods to investigated the half-lives of WT and variants. 0.1 mg/mL enzyme was incubated with or without CL (0.125, 0.25 and 0.5 mM) in the assay buffer. The enzyme activities were determined by adding 15 nM of pre-incubated enzyme according to Section 4.3 after various incubation times up to 60 min at an assay temperature of 25 °C. Experiments were done at least three times independently as double determination.

### 4.5. Assessment of Protein Stability

The protein stability was investigated applying nano differential scanning fluorimetry (nanoDSF) using a protein concentration of 1 mg/mL in assay buffer. The influence of CL on the protein stability was investigated by adding CL to the protein containing samples with final concentration of 0.25 mM. The heating rate has been set to 1 °C per min. A heating ramp from 15 to 95 °C was determined. The nanoDSF data were obtained using a Prometheus NT.48 device. First derivatives were calculated using PR.ThermControl software (NanoTemper Technologies GmbH, Munich, Germany). Experiments were performed at least three times independently as double determination.

### 4.6. Determination of the Kinetic Parameters

It is assumed that the reaction catalyzed by CPT II represents a ping-pong mechanism or compulsory order ternary-complex mechanism [43,44]. K_M_ and V_max_ values for palmitoyl-CoA or L-carnitine were determined using concentration series of Pal-CoA (0–150 µM) or L-carnitine (0–200 mM), a constant amount of the other CPT II substrate (30 nM Pal-CoA, 12 mM carnitine) and 7 nM enzyme. These measurements were also performed in the presence of CL (0.25 mM) to investigate the effect of CL on the kinetic parameters of CPT II.

The kinetic parameters were determined by evaluating the obtained v/S characteristic according to the Michaelis–Menten Equation (1). The catalytic efficiency *k_cat_* was calculated using Equation (2):

(1) $$v=\frac{V_{\max}*\left[S\right]}{K_{M}+\left[S\right]}$$

(2) $$k_{\mathrm{cat}}=\frac{V_{\max}}{c_{\mathrm{enzyme}}}$$

Experiments were performed at least three times independently as double determination.

### 4.7. Determination of Inhibition Constant of for R-amino Carnitine for CPT II

The inhibition constant K_i_ for the competitive inhibitor R-amino carnitine was determined using various concentrations (R-AC, 0, 0.25 and 0.5 mM). A concentration series of L-carnitine in the enzyme assay was used (0–200 mM) while the concentration of Pal-CoA remained constant at 30 µM. Experiments were performed at least three times independently as double determination.

The values for K_i_ were calculated on the basis of the obtained K_M_ values, the Michaelis–Menten constant of the inhibited reaction K_Mi_ and the inhibitor concentration using the following equation [45]:

(3) $$K_{\mathrm{Mi}}=\left(1+\frac{\left[I\right]}{\;K_{i}}\right)K_{M}$$

(4) $$K_{i}=\frac{K_{M}\cdot \left[I\right]}{K_{\mathrm{Mi}}-K_{M}}$$

### 4.8. Lipid Binding Assay

Direct interaction of CPT II WT and variants with phospholipids of the mitochondrial membrane was assessed using membrane lipid strips (Echelon Biosciences, Salt Lake City, Utah, USA). The membrane was spotted with 100 pmol of various cell membrane lipids. The assay was performed following manufacturer’s instructions with some modifications explained in the following. At first, the membrane was washed twice in phosphate buffered saline (PBS). After 1 h incubation with blocking solution (3% BSA fatty acid-free in PBS) at room temperature, the membrane was incubated with purified CPT II WT or variant (final amount 0.4 mg protein) for 2 h at 4 °C. After three washing steps, a-His-Probe (H3) mouse monoclonal antibody (Santa Cruz Biotech, Santa Cruz, CA, USA, 1:1000 dilution in PBS containing 3% BSA) was added to the membrane over night at 4 °C. The membrane was washed three times with PBS and incubated with mouse IgG horseradish peroxidase-linked whole antibody (from sheep, Amersham, Little Chalfont, Buckinghamshire, UK) for 1 h at 4 °C. The interaction was visualized by ECL Western blotting detection reagents (Amersham, Little Chalfont, Buckinghamshire, UK).

### 4.9. Statistical Analysis

Statistical analysis and calculation were performed using Prism 8.3 (GraphPad, San Diego, CA, USA). An analysis was done using ordinary one-way ANOVA with Bonferroni’s multiple-comparison test. The level of significance was set to *p* < 0.05.

## 5. Conclusions

The amino acid exchanges in CPT II variants investigated here, namely S113L, P50H and Y479F, resulted in the structural destabilization of the enzyme, whereby the variants differed strongly regarding their thermostability. These amino acid exchanges had no impact on the specific enzyme activity of the variants compared to WT. The phospholipid CL stabilized WT and the variants and significantly increased the catalytic efficiency for the second substrate L-carnitine by improving substrate binding. In general, the mutations that lead to the destabilization of CPT II are associated with the muscular form of CPT II deficiency. This is independent of the differences in the thermosensitivity of the variants. Despite various stabilization options (e.g., CL), this might not sufficiently prevent the symptoms of CPT II deficiency.

## Figures and Tables

**Table 1:** Effect of 0.25 mM CL on the kinetic parameters *k*_cat_ and K_M_ and the catalytic efficiency of CPT II WT and variants for palmitoyl-CoA as varying substrate at 25 °C (*n* ≥ 3).

CPT II Variant	In Absence of CL			In Presence of CL		
	*k*_cat_ (s^−1^)	K_M_ (µM)	*k*_cat_/K_M_ (µM^1^ s^−1^)	*k*_cat_ (s^−1^)	K_M_ (µM)	*k*_cat_/K_M_ (µM^1^ s^−1^)
WT	144.7 ± 3.1	7.10 ± 1.1	20.5 ± 3.9	145.3 ± 9.0	6.7 ± 1.1	21.8 ± 2.3
P50H	144.3 ± 18	5.50 ± 1.4	26.4 ± 7.5	138.2 ± 8.4	6.7 ±1.8	20.7 ± 3.8
S113L	142.1 ± 25	11.0 ± 3.6	12.9 ± 1.8 *	145.8 ± 0.9	12.3 ± 3.0	11.9 ± 2.9 *
Y479F	141.4 ± 1.7	5.10 ± 2.3	27.5 ± 4.4	144.8 ± 13	6.7 ± 0.3	21.7 ± 4.7

* indicates a significant difference from WT to variant, *p* < 0.05.

**Table 2:** Effect of 0.25 mM CL on the kinetic parameters *k*_cat_ and K_M_ and the catalytic efficiency of CPT II WT and variants for L-carnitine as varying substrate at 25 °C (*n* ≥ 3).

CPT II Variant	In Absence of CL			In Presence of CL		
	*k*_cat_ (s^−1^)	K_M_ (mM)	*k*_cat_/K_M_ (mM^1^ s^−1^)	*k*_cat_ (s^−1^)	K_M_ (mM)	*k*_cat_/K_M_ (mM^1^ s^−1^)
WT	148.6 ± 11.1	15.1 ± 3.4	9.8 ± 1.6	129.9 ± 8.1	5.5 ± 1.0	23.6 ± 3.1 *
P50H	137.7 ± 4.9	12.8 ± 2.7	10.8 ± 2.6	138.1 ± 5.6	6.6 ±2.8	21.0 ± 1.8 *
S113L	144.0 ± 5.0	15.5 ± 2.3	9.3 ± 1.4	135.2 ± 2.5	6.5 ± 0.2	20.7 ± 0.3 *
Y479F	140.5 ± 18.5	17.4 ± 4.4	8.1 ± 1.1	138.7 ± 1.4	4.5 ± 0.5	30.9 ± 2.6 *

* indicates a significant difference from *k*_cat_/K_M_ in absence of CL, *p* < 0.05.

**Table 3:** Effect of 0.25 mM CL on the inhibition constant K_i_ for the inhibitor R-amino carnitine at 25 °C (*n* ≥ 3).

CPT II Variant	Inhibition Constant K_i_ (mM) in Absence of CL	Inhibition Constant K_i_ (mM) in Presence of CL
WT	0.493 ± 0.004	0.105 ± 0.002 **
P50H	0.134 ± 0.011	0.046 ± 0.006 **
S113L	0.173 ± 0.020	0.036 ± 0.016 **
Y479F	0.756 ± 0.062	0.108 ± 0.039 **

** indicates significant difference from K_i_ in absence of CL, *p* < 0.005.

**Table 4:** Primer sequences for site directed mutagenesis. The nucleotide triplets of the amino acid exchanges of the respective variant are labelled green.

CPT II Variant	Sense Sequence (5′-3′)	Antisense Sequence (5′-3′)
P50H	GGACAGCCTG**CAT**AGGCTGCCTATTC	TGGTAGTGCATGGTGGGC
S113L	AAGCTACATT**TTG**GGACCCTGGT	GTATGTTTATTCTGTTTGTCCAGAG
Y479F	CTGCGGCAG**TTT**GGGCAGACAG	GAAGGCCATCTGGAATGC

## Data Availability

The datasets generated and analyzed during the current study are available from the corresponding author on reasonable request.

## Supplementary Materials

Supplementary materials can be found at https://www.mdpi.com/article/10.3390/ijms22094831/s1, Figure S1: Scattered light back reflection as measure for aggregation during the heating ramp from 15 °C to 80 °C of CPT II WT (black) and the variants P50H (red), S113L (blue) and Y479F (green) in assay buffer (magenta). The light scattering data were obtained simultaneously with nanoDSF data using the back reflection technology in a Prometheus NT.48 device. The unfolding of proteins leads to display of hydrophobic regions of the protein on the surface, which is followed by hydrophobic interaction of the protein molecules resulting in aggregation events. These aggregates are then detected via light scattering. The curve trajectories for the colloidal stability of CPT II show that at the beginning of the thermal transition no aggregation was detectable. The data allow the detection of onset of aggregation (T_agg_) which differs clearly among the variants. The T_agg_ for WT is 38 °C, Y479F is 37 °C, S113L is 35 °C and P50H is 32 °C. These results support the hypothesis of increases thermosensitivity of the variants. Figure S2: The first derivatives of the transition curves of WT and the variants resulting from the ratio 350/330 nm were plotted against the temperature. The black, red, blue and green solid lines show the curves of WT and variants P50H, S113L and Y479F measured in assay buffer, respectively. The black, red, blue and green dashed lines show the maxima of the values of the first derivatives of the ratio 350 nm/330 nm of WT and variants P50H, S113L and Y479F, respectively. Figure S3: The effect of amino acid exchange in absence and presence of n-octyl-β-d-glucopyranoside (β-OG) on the stability of CPT II (1 mg/mL) assessed by nanoDSF. The T_M_ of WT (37 °C) in buffer was used as reference value (ΔT_M_ = 0 °C) to determine the ΔT_M_ for the variants and the ΔT_M_ for WT and variants in presence of β- OG. Error bars are SD derived from three independent experiments. Table S1. Comparison of the mean values of the half-lives (t_1/2_) of CPT II WT and the variants at 30 °C with the half-lives at 37, 40 or 42 °C. The differences of the half-lives for each CPT II enzyme between 37 and 40 or 42 °C as well as 40 and 42 °C are not significant. Number of experiments for each variant (*n* ≥ 3). Table S2. Comparison of the mean values of the half-lives (t_1/2_) between CPT II WT and the variants Y479F, S113L and P50H at 30, 37, 40 or 42 °C. *n* ≥ 3. Table S3. Comparison of the maximum activities of CPT II WT and the variants Y479F, S113L and P50H in buffer with maximum enzymatic activities in presence of 0.25 or 0.5 mM cardiolipin (*n* ≥ 3). Table S4. Comparison of the maximum activities between CPT II WT and the variants Y479F, S113L or P50H in buffer with of 0.25 or 0.5 mM cardiolipin (*n* ≥ 3). Table S5. Comparison of the ∆T_M_-values of CPT II WT and the variants Y479F, S113L and P50H in buffer or in presence of 0.25 mM cardiolipin or 1% n-octyl-β-d-glucopyranoside (β-OG) assessed by nanoDSF (*n* ≥ 3). Table S6. Comparison of the transition temperatures (T_M_) of WT and the variants Y479F, S113L and P50H in buffer with of 0.25 mM cardiolipin or 1% n-octyl-β-d-glucopyranoside (β-OG) assessed by nanoDSF (*n* ≥ 3). Since the differences of the T_M_ values between buffer with 0.25 mM CL and buffer with 1% β-OG are not significant, these data are not shown. Table S7. Comparison of the transition temperatures between WT and the variants Y479F, S113L or P50H in buffer, in presence of 0.25 mM cardiolipin or 1% n-octyl-β-d-glucopyranoside (β-OG) (*n* ≥ 3).

## Abbreviations

CPT	Carnitine palmitoyltransferase
CACT	Carnitine:acylcarnitine translocase
LCFA	Long chain fatty acid
CL	Cardiolipin
Pal-CoA	Palmitoyl-Coenzyme A
CoA	Coenzyme A
WT	Wild type
PBS	Phosphate buffered saline
R-AC	R-Aminocarnitine
IMM	Inner mitochondrial membrane
β-OG	n-octyl-β-d-glucopyranoside
nanoDSF	Nano differential scanning fluorimetry

## References

[1] Houten S.M., Wanders R.J. (2010). A General Introduction to the Biochemistry of Mitochondrial Fatty Acid Beta-Oxidation. J. Inherit. Metab. Dis..

[2] McGarry J.D., Brown N.F. (1997). The Mitochondrial Carnitine Palmitoyltransferase System. From Concept to Molecular Analysis. Eur. J. Biochem..

[3] McGarry J. (2001). Travels with Carnitine Palmitoyltransferase I: From Liver to Germ Cell with Stops in Between.

[4] Finocchiaro G., Colombo I., DiDonato S. (1990). Purification, Characterization and Partial Amino Acid Sequences of Carnitine Palmitoyl-Transferase from Human Liver. FEBS Lett..

[5] Roe C., Coates P., Scriver C., Beaudet A.L., Sly W.S., Valle D. (1995). Disorders of Mitochondrial Function. The Metabolic and Molecular Ba Inherited Disease.

[6] Console L., Giangregorio N., Indiveri C., Tonazzi A. (2014). Carnitine/Acylcarnitine Translocase and Carnitine Palmitoyltransferase 2 form a Complex in the Inner Mitochondrial Membrane. Mol. Cell. Biochem..

[7] Rufer A.C., Thoma R., Hennig M. (2009). Structural Insight into Function and Regulation of Carnitine Palmitoyltransferase. Cell Mol. Life Sci..

[8] Osman C., Voelker D.R., Langer T. (2011). Making Heads or Tails of Phospholipids in Mitochondria. J. Cell Biol..

[9] Paradies G., Paradies V., De Benedictis V., Ruggiero F.M., Petrosillo G. (2014). Functional Role of Cardiolipin in Mitochondrial Bioenergetics. Biochim. Biophys. Acta.

[10] Ikon N., Ryan R.O. (2017). Cardiolipin and Mitochondrial Cristae Organization. Biochim. Biophys. Acta Biomembr..

[11] Kagan V.E., Bayir H.A., Belikova N.A., Kapralov O., Tyurina Y.Y., Tyurin V.A., Jiang J., Stoyanovsky D.A., Wipf P., Kochanek P.M. (2009). Cytochrome C/Cardiolipin Relations in Mitochondria: A Kiss of Death. Free Radic. Biol. Med..

[12] Schlame M., Rua D., Greenberg M.L. (2000). The biosynthesis and functional role of cardiolipin. Prog. Lipid Res..

[13] Noël H., Pande S.V. (1986). An Essential Requirement of Cardiolipin for Mitochondrial Carnitine Acylcarnitine Translocase Activity: Lipid Requirement of Carnitine Acylcarnitine Translocase. Eur. J. Biochem..

[14] Rubio-Gozalbo M., Bakker J., Waterham H., Wanders R. (2004). Carnitine–Acylcarnitine Translocase Deficiency, Clinical, Biochemical and Genetic Aspects. Mol. Asp. Med..

[15] Palsdottir H., Hunte C. (2004). Lipids in Membrane Protein Structures. Biochim. Biophys. Acta.

[16] Rufer A.C., Lomize A., Benz J., Chomienne O., Thoma R., Hennig M. (2007). Carnitine Palmitoyltransferase 2: Analysis of Membrane Association and Complex Structure with a Substrate Analog. FEBS Lett..

[17] Kashfi K., Mynatt R.L., Park E.A., Cook G.A. (2011). Membrane Microenvironment Regulation of Carnitine Palmitoyltranferases I and II. Biochem. Soc. Trans..

[18] Isackson P.J., Bennett M.J., Lichter-Konecki U., Willis M., Nyhan W.L., Sutton V.R., Tein I., Vladutiu G.D. (2008). CPT2 Gene Mutations Resulting in Lethal Neonatal or Severe Infantile Carnitine Palmitoyltransferase II Deficiency. Mol. Genet. Metab..

[19] DiMauro S., DiMauro P.M.M. (1973). Muscle Carnitine Palmityltransferase Deficiency and Myoglobinuria. Science.

[20] Bonnefont J.P., Djouadi F., Prip-Buus C., Gobin S., Munnich A., Bastin J. (2004). Carnitine Palmitoyltransferases 1 and 2: Biochemical, Molecular and Medical Aspects. Mol. Asp. Med..

[21] Zierz S. (1994). Carnitine Palmitoyltransferase Deficiency. Myopathy.

[22] Deschauer M., Wieser T., Zierz S. (2005). Muscle Carnitine Palmitoyltransferase II Deficiency: Clinical and Molecular Genetic Features and Diagnostic Aspects. Arch. Neurol..

[23] Joshi P.R., Deschauer M., Zierz S. (2014). Carnitine Palmitoyltransferase II (CPT II) Deficiency: Genotype-Phenotype Analysis of 50 Patients. J. Neurol. Sci..

[24] Nielsen M. (1938). Die Regulation der Körpertemperatur bei Muskelarbeit 1. Skand. Arch. Für Physiol..

[25] PELLEGRINI A., RIVA G., Margaria R. (1947). La Termoregolazione Nel Lavoro Muscolare. Arch. Di. Fisiol..

[26] Jameson J.L. (2018). Harrison’s Principles of Internal Medicine.

[27] Kluger M.J. (2015). Fever: Its Biology, Evolution, and Function.

[28] Chung N., Park J., Lim K. (2017). The Effects of Exercise and Cold Exposure on Mitochondrial Biogenesis in Skeletal Muscle and White Adipose Tissue. J. Exerc. Nutr. Biochem..

[29] Motlagh L., Golbik R., Sippl W., Zierz S. (2016). Stabilization of the Thermolabile Variant S113L of Carnitine Palmitoyltransferase II. Neurol. Genet..

[30] Rufer A.C., Thoma R., Benz J., Stihle M., Gsell B., De Roo E., Banner D.W., Mueller F., Chomienne O., Hennig M. (2006). The Crystal Structure of Carnitine Palmitoyltransferase 2 and Implications for Diabetes Treatment. Structure.

[31] Isackson P.J., Bennett M.J., Vladutiu G.D. (2006). Identification of 16 New Disease-Causing Mutations in the CPT2 Gene Resulting in Carnitine Palmitoyltransferase II deficiency. Mol. Genet. Metab..

[32] Motlagh L., Golbik R., Sippl W., Zierz S. (2016). Malony-CoA Inhibits the S113L Variant of Carnitine-Palmitoyltransferase II. Biochim. Biophys. Acta.

[33] Verderio E., Cavadini P., Montermini L., Wang H., Lamantea E., Finocchiaro G., DiDonato S., Gellera C., Taroni F. (1995). Carnitine Palmitoyltransferase II Deficiency: Structure of the Gene and Characterization of Two Novel Disease-Causing Mutations. Hum. Mol. Genet..

[34] Wieser T., Deschauer M., Olek K., Hermann T., Zierz S. (2003). Carnitine Palmitoyltransferase II Deficiency: Molecular and Biochemical Analysis of 32 Patients. Neurology.

[35] Hsiao Y.-S., Jogl G., Esser V., Tong L. (2006). Crystal Structure of Rat Carnitine Palmitoyltransferase II (CPT-II). Biochem. Biophys. Res. Commun..

[36] Wen J., Lord H., Knutson N., Wikstrom M. (2020). Nano Differential Scanning Fluorimetry for Comparability Studies of Therapeutic Proteins. Anal. Biochem..

[37] Gihaz S., Weiser D., Dror A., Satorhelyi P., Jerabek-Willemsen M., Poppe L., Fishman A. (2016). Creating an Efficient Methanol-Stable Biocatalyst by Protein and Immobilization Engineering Steps towards Efficient Biosynthesis of Biodiesel. ChemSusChem.

[38] Taroni F., Verderio E., Dworzak F., Willems P.J., Cavadini P., DiDonato S. (1993). Identification of a common mutation in the carnitine palmitoyltransferase II gene in familial recurrent myoglobinuria patients. Nat. Genet..

[39] Yasuno T., Kaneoka H., Tokuyasu T., Aoki J., Yoshida S., Takayanagi M., Ohtake A., Kanazawa M., Ogawa A., Tojo K. (2008). Mutations of Carnitine Palmitoyltransferase II (CPT II) in Japanese Patients with CPT II Deficiency. Clin. Genet..

[40] Yao D., Mizuguchi H., Yamaguchi M., Yamada H., Chida J., Shikata K., Kido H. (2008). Thermal Instability of Compound Variants of Carnitine Palmitoyltransferase II and Impaired Mitochondrial Fuel Utilization in Influenza-Associated Encephalopathy. Hum. Mutat..

[41] Motlagh Scholle L., Thaele A., Beckers M., Meinhardt B., Zierz S. (2018). Lack of Activation of the S113L Variant of Carnitine Palmitoyltransfersase II by Cardiolipin. J. Bioenerg. Biomembr..

[42] Ellman G.L. (1958). A Colorimetric Method for Determining Low Concentrations of Mercaptans. Arch. Biochem. Biophys..

[43] Nic a′ Bháird N., Kumaravel G., Gandour R., Krueger M., Ramsay R. (1993). Comparison of the Active Sites of the Purified Carnitine Acyltransferases from Peroxisomes and Mitochondria by Using a Reaction-Intermediate Analogue. Biochem. J..

[44] Brown N.F., Anderson R.C., Caplan S.L., Foster D.W., McGarry J.D. (1994). Catalytically Important Domains of Rat Carnitine Palmitoyltransferase II as Determined by Site-Directed Mutagenesis and Chemical Modification. Evidence for a Critical Histidine Residue. J. Biol. Chem..

[45] Copeland R.A. (2005). Evaluation of Enzyme Inhibitors in Drug Discovery. Wiley-Interscience.

