# Supplementary material for: Cardiolipin Stabilizes and Increases Catalytic Efficiency of Carnitine Palmitoyltransferase II and Its Variants S113L, P50H, and Y479F
Source: Int J Mol Sci. 2021 May 2;22(9):4831. doi: 10.3390/ijms22094831 (PMC8125234; doi:10.3390/ijms22094831)
Supplement: Supplementary file 1 [file ijms-22-04831-s001.zip › ijms-1177673-supplementary.pdf]

International Journal of *Molecular Sciences*

Supplementary Materials:

Cardiolipin stabilizes and increases catalytic efficiency of carnitine palmitoyltransferase II and its variants S113L, P50H, and Y479F

Beate Meinhardt, Leila Motlagh Scholle, Franziska Seifert, Martina Anwand, Markus Pietzsch and Stephan Zierz

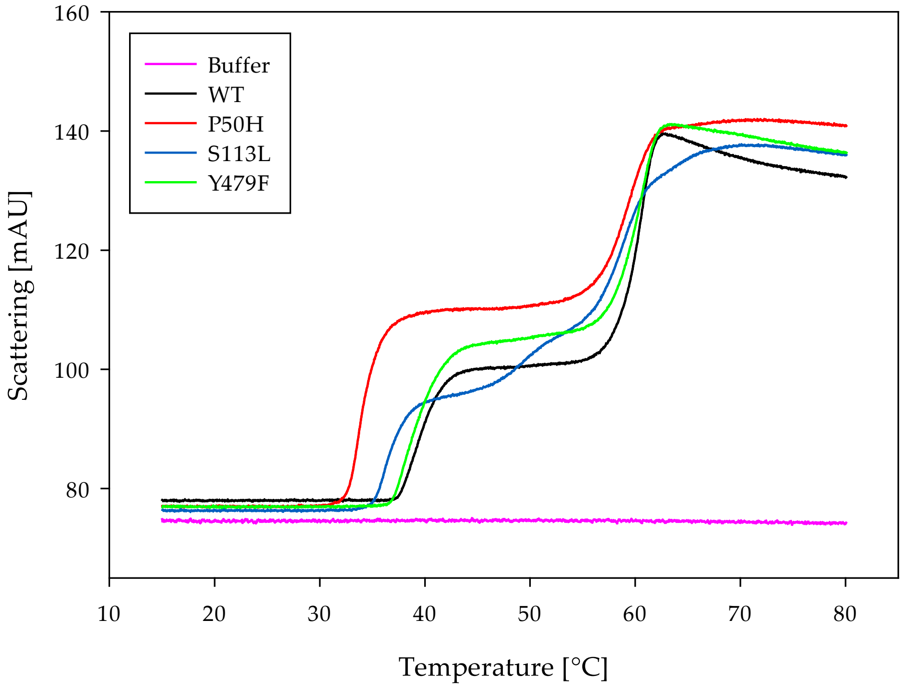

Figure S1: Scattered light back reflection as measure for aggregation during the heating ramp from 15 °C to 80 °C of CPT II WT (black) and the variants P50H (red), S113L (blue) and Y479F (green) in assay buffer (magenta). The light scattering data were obtained simultaneously with nanoDSF data using the back reflection technology in a Prometheus NT.48 device. The unfolding of proteins leads to display of hydrophobic regions of the protein on the surface and this is followed by hydrophobic interaction of the protein molecules resulting in aggregation events. These aggregates are then detected via light scattering. The curve trajectories for the colloidal stability of CPT II show that at the beginning of the thermal transition no aggregation was detectable. The data allow the detection of onset of aggregation (T_{agg}) which differs clearly among the variants. The T_{agg} for WT is 38 °C, Y479F 37 °C, S113L 35 °C and P50H 32 °C. These results support the hypothesis of increases thermosensitivity of the variants.

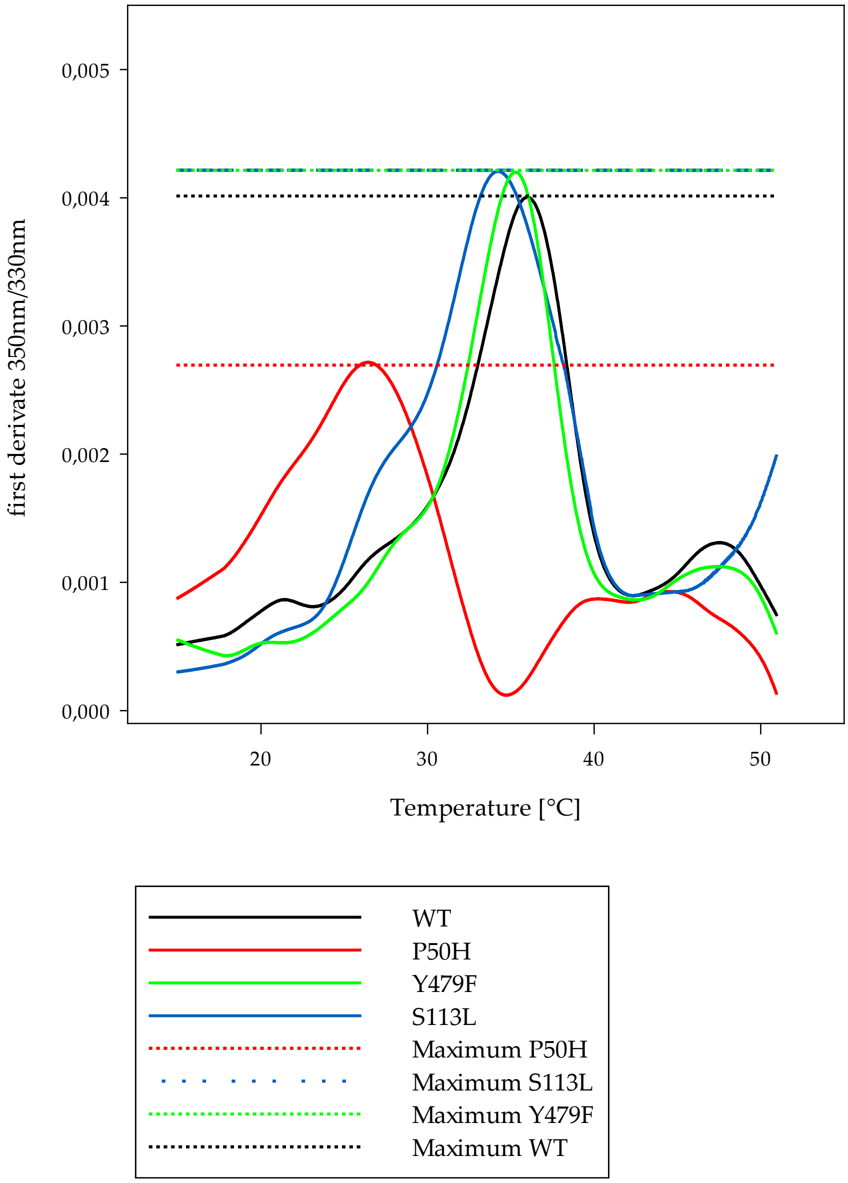

Figure S2: First derivatives of the transition curves of WT and the variants resulting from the ratio 350/330 nm were plotted against the temperature. The black, red, blue and green solid lines show the curves of WT and variants P50H, S113L and Y479F measured in assay buffer, respectively. The black, red, blue and green dashed lines show the maxima of the values of the first derivatives of the ratio 350 nm/330 nm of WT and variants P50H, S113L and Y479F, respectively.

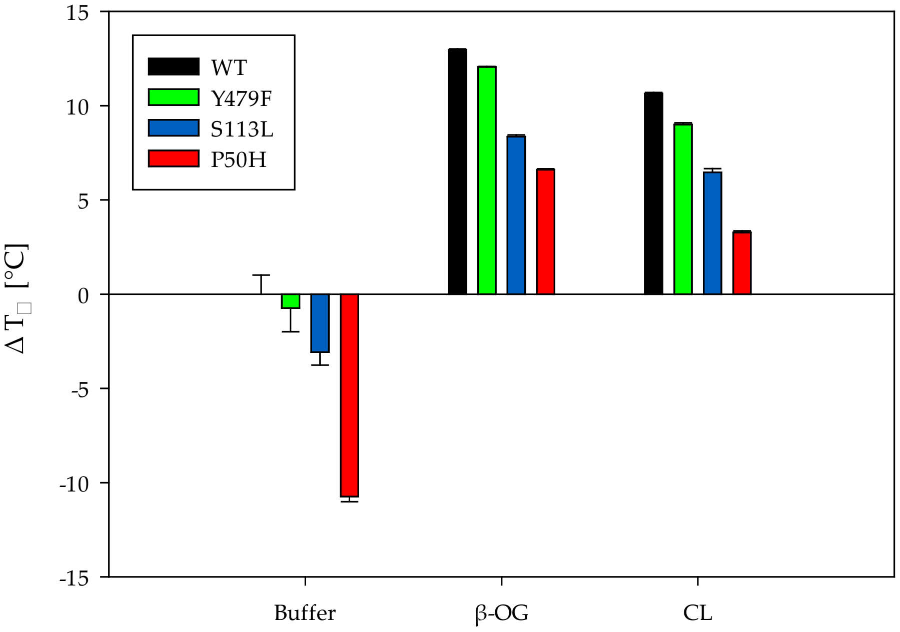

Figure S3: The effect of amino acid exchange in absence and presence of n-octyl-β-d-glucopyranoside (β-OG, 1 %) or the addition of CL (25 mM) on the stability of CPT II (1 mg/mL) assessed by nanoDSF. The T_M of WT (37 °C) in buffer served as reference value (ΔT_M = 0 °C) to determine the ΔT_M for the variants in buffer. This reference value was also used to determine the ΔT_M for WT and the variants in presence of β- OG or CL. Error bars are SD derived from three independent experiments. The significant differences are shown in Table S3-S5.

Table S1. Comparison of the mean values of the half-lives ($t_{1/2}$) of CPT II WT and the variants at 30 °C with the half-lives at 37 °C, 40 °C or 42 °C. The differences of the half-lives for each CPT II enzyme between 37 °C and 40 °C or 42 °C as well as 40 °C and 42 °C are not significant. Number of experiments for each variant (n) ≥ 3.

	$t_{1/2}$ (min)						
**CPT II Variant**	**30 °C**	**37 °C**	***p*-value**	**40 °C**	***p*-value**	**42 °C**	**p-value**
WT	533.2 ± 168.4	7.9 ± 0.4	<0.0001	2.0 ± 0.1	<0.0001	0.7 ± 0.1	<0.0001
Y479F	121.6 ± 25.3	2.1 ± 0.1	<0.0001	0.6 ± 0.02	<0.0001	0.3 ± 0.02	<0.0001
S113L	27.5 ± 1.2	1.2 ± 0.1	<0.0001	0.4 ± 0.01	<0.0001	0.3 ± 0.01	<0.0001
P50H	3.3 ± 0.1	0.3 ± 0.01	<0.0001	0.2 ± 0.004	<0.0001	0.2 ± 0.003	<0.0001

International Journal of *Molecular Sciences*

MDPI

Table S2. Comparison of the mean values of the half-lives ($t_{1/2}$) between CPT II WT and the variants Y479F, S113L and P50H at 30 °C, 37 °C, 40 °C or 42 °C. n ≥ 3

**$t_{1/2}$ (min) at 30 °C**								
		***p*-value**			***p*-value**			***p*-value**
	Y479F 121.6 ± 25.3	0.0001		S113L 27.5 ± 1.2	0.8641			
WT 533.2 ± 168.4	S113L 27.5 ± 1.2	<0.0001	Y479F 121.6 ± 25.3	P50H 3.3 ± 0.1	0.4377	S113L 27.5 ± 1.2	P50H 3.3 ± 0.1	>0.9999
	P50H 3.3 ± 0.1	<0.0001						
**$t_{1/2}$ (min) at 37 °C**								
		***p*-value**			***p*-value**			***p*-value**
	Y479F 2.1 ± 0.1	<0.0001		S113L 1.2 ± 0.1	0.0004			
WT 7.9 ± 0.4	S113L 1.2 ± 0.1	<0.0001	Y479F 2.1 ± 0.1	P50H 0.3 ± 0.01	<0.0001	S113L 1.2 ± 0.1	P50H 0.3 ± 0.01	0.0002
	P50H 0.3 ± 0.01	<0.0001						
**$t_{1/2}$ (min) at 40 °C**								
		***p*-value**			***p*-value**			***p*-value**
	Y479F 0.6 ± 0.02	<0.0001		S113L 0.4 ± 0.01	<0.0001			
WT 2.0 ± 0.1	S113L 0.4 ± 0.01	<0.0001	Y479F 0.6 ± 0.02	P50H 0.2 ± 0.004	<0.0001	S113L 0.4 ± 0.01	P50H 0.2 ± 0.004	0.0003
	P50H 0.2 ± 0.004	<0.0001						
**$t_{1/2}$ (min) at 42 °C**								
		***p*-value**			***p*-value**			***p*-value**
	Y479F 0.3 ± 0.02	<0.0001		S113L 0.3 ± 0.01	>0.9999			
WT 0.7 ± 0.1	S113L 0.3 ± 0.01	<0.0001	Y479F 0.3 ± 0.02	P50H 0.2 ± 0.003	0.0002	S113L 0.3 ± 0.01	P50H 0.2 ± 0.003	0.0005
	P50H 0.2 ± 0.003	<0.0001						

International Journal of Molecular Sciences

MDPI

Table S3. Comparison of the maximum enzymatic activities of CPT II WT and the variants Y479F, S113L and P50H in buffer with maximum enzymatic activities in presence of 0.25 mM or 0.5 mM cardiolipin (n ≥ 3).

	Maximal Enzymatic Activity (%)				
**CPT II Variant**	**w/o CL**	**0.25 mM CL**	***p*-value**	**0.5 mM CL**	***p*-value**
WT	100 ± 5.1	152.3 ± 5.5	<0,0001	135.9 ± 4.7	<0,0001
Y479F	100 ± 4.8	175.2 ± 2,8	<0,0001	151.7 ± 7.6	<0,0001
S113L	100 ± 7.4	114.5 ± 8.3	0,0007	98.7 ± 5.1	0,9179
P50H	100 ± 3.9	113.0 ± 7.6	0,0024	94.5 ± 6.4	0,2599

Table S4. Comparison of the maximum enzymatic activities between CPT II WT and the variants Y479F, S113L or P50H in buffer with of 0.25 mM or 0.5 mM cardiolipin (n ≥ 3).

Maximal Enzymatic Activity (%)					
**0.25 mM CL**			**0.5 mM CL**		
		***p*-value**			***p*-value**
WT 152.3 ± 5.5	Y479F 175.2 ± 2,8	0,0009	WT 135.9 ± 4.7	Y479F 151.7 ± 7.6	0,01
	S113L 114.5 ± 8.3	<0,0001		S113L 98.7 ± 5.1	<0,0001
	P50H 113.0 ± 7.6	<0,0001		P50H 94.5 ± 6.4	<0,0001

Table S5. Comparison of the ΔT_M –values of CPT II WT and the variants Y479F, S113L and P50H in buffer or in presence of 0.25 mM cardiolipin or 1 % n-octyl-β-d-glucopyranoside (β-OG) assessed by nanoDSF (n ≥ 3).

CPT II Variant	ΔT_M in Buffer	ΔT_M in Buffer with 0.25 mM CL	ΔT_M in Buffer with 1 % β-OG	*p*-value
WT	0.0 ± 1.0	10.6 ± 0.6	13.0 ± 0.7	<0.0001
Y479F	-0.74 ± 1.2	9.0 ± 0.1	12.1 ± 0.5	<0.0001
S113L	-3.1 ± 0.7	6.5 ± 0.2	8.4 ± 0.6	<0.0001
P50H	-10.7 ± 0.3	3.3 ± 0.1	3.3 ± 0.4	<0.0001

Table S6. Comparison of the transition temperatures (T_M) of WT and the variants Y479F, S113L and P50H in buffer with of 0.25 mM cardiolipin or 1 % n-octyl-β-d-glucopyranoside (β-OG) assessed by nanoDSF (n ≥ 3). Since the differences of the T_M values between buffer with 0.25 mM CL and buffer with 1 % β-OG are not significant, these data are not shown.

CPT II Variant	T_M in Buffer	T_M in Buffer with 0.25 mM CL	*p*-value	T_M in Buffer with 1 % β-OG	*p*-value
WT	36.9 ± 1.0	47.6 ± 1.3	<0.0001	49.9 ± 0.7	<0.0001
Y479F	36.2 ± 1.2	45.9 ± 1.0	<0.0001	49.0 ± 0.5	<0.0001
S113L	33.8 ± 0.7	43.4 ± 0.8	<0.0001	45.3 ± 0.6	<0.0001
P50H	26.2 ± 0.3	40.2 ± 0.7	<0.0001	43.5 ± 0.4	<0.0001

International Journal of *Molecular Sciences*

MDPI

Table S7. Comparison of the transition temperatures between WT and the variants Y479F, S113L or P50H in buffer, in presence of 0.25 mM cardiolipin or 1 % n-octyl-β-d-glucopyranoside (β-OG), respectively (n ≥ 3).

**Buffer**				**Buffer with 0.25 mM CL**				**Buffer with 1 % β-OG**			
		**T_M (°C)**	***p*-value**			**T_M (°C)**	***p*-value**			**T_M (°C)**	***p*-value**
WT 36.9 ± 1.0	Y479F	36.2 ± 1.2	0.0690	WT 47.6 ± 1.3	Y479F	45.9 ± 1.0	0.0183	WT 49.9 ± 0.7	Y479F	49.0 ± 0.5	0.1926
	S113L	33.8 ± 0.7	<0.0001		S113L	43.4 ± 0.8	<0.0001		S113L	45.3 ± 0.6	< 0.0001
	P50H	26.2 ± 0.3	<0.0001		P50H	40.2 ± 0.7	<0.0001		P50H	43.5 ± 0.4	< 0.0001
